# Chromosome-level genome assembly of Korean holoparasitic plants*, Orobanche coerulescens*

**DOI:** 10.1038/s41597-024-03207-1

**Published:** 2024-07-02

**Authors:** Bongsang Kim, So Yun Jhang, Bomin Koh, Soonok Kim, Won-Jae Chi, Jeong-Mi Park, Chae Eun Lim, Yoonjee Hong, Heebal Kim, Jaewoong Yu, Seoae Cho

**Affiliations:** 1 Department of Agricultural Biotechnology and Research Institute of Agriculture and Life Sciences, Seoul National University, Seoul, Republic of Korea; 2 eGnome, Inc, Seoul, Republic of Korea; 3 Interdisciplinary Program in Bioinformatics, Seoul National University, Seoul, Republic of Korea; 4 National Institute of Biological Resources, Incheon, Republic of Korea

**Keywords:** Plant evolution, Evolutionary genetics

## Abstract

*Orobanche coerulescens* is a parasitic plant that cannot complete its life cycle without a host and is incapable of photosynthesis. The habitats of *O. coerulescens* span the coasts of Korea and its volcanic islands, Ulleungdo and Dokdo. Those on the volcanic islands exhibit morphological differences and have distinct hosts compared to those on the peninsula. The family of Orobanchaceae, encompassing both autotrophic and parasitic species, serves as a model for evolutionary studies of parasitic states. However, there are limited genome assemblies for the *Orobanche* genus. In our study, we produced approximately 100x ONT long reads to construct a chromosome-level genome of *O. coerulescens*. The resulting assembly has a total size of 3,648 Mb with an N50 value of 195 Mb, and 82.0% of BUSCO genes were identified as complete. Results of the repeat annotation revealed that 86.3% of the genome consisted of repeat elements, and 29,395 protein-coding genes were annotated. This chromosome-level genome will be an important biological resource for conserving biodiversity and further understanding parasitic plants.

## Background & Summary

*Orobanche coerulescens*, a member of the Orobanchaceae family, is a holoparasitic plant with an obligate life cycle. Approximately 4,750 species of angiosperms are parasitic, relying wholly or partially on their host plants for water and nutrients to sustain their lives^1^. Parasitic plants can be classified based on their reliance on hosts^2–4^. Obligate parasites necessitate a host to complete their life cycle, while facultative ones do not. Hemiparasites are capable of photosynthesis but obtain water, minerals, and nutrients from their hosts. On the other hand, holoparasites lack chlorophyll and derive their fixed carbon from their hosts, presenting non-green colours. The Orobanchaceae family encompasses diverse autotrophic genera as well as hemiparasitic and holoparasitic lineages, providing an ideal model for evolutionary studies given its comprehensive range of parasitic states^5–7^.

**Fig. 1:**
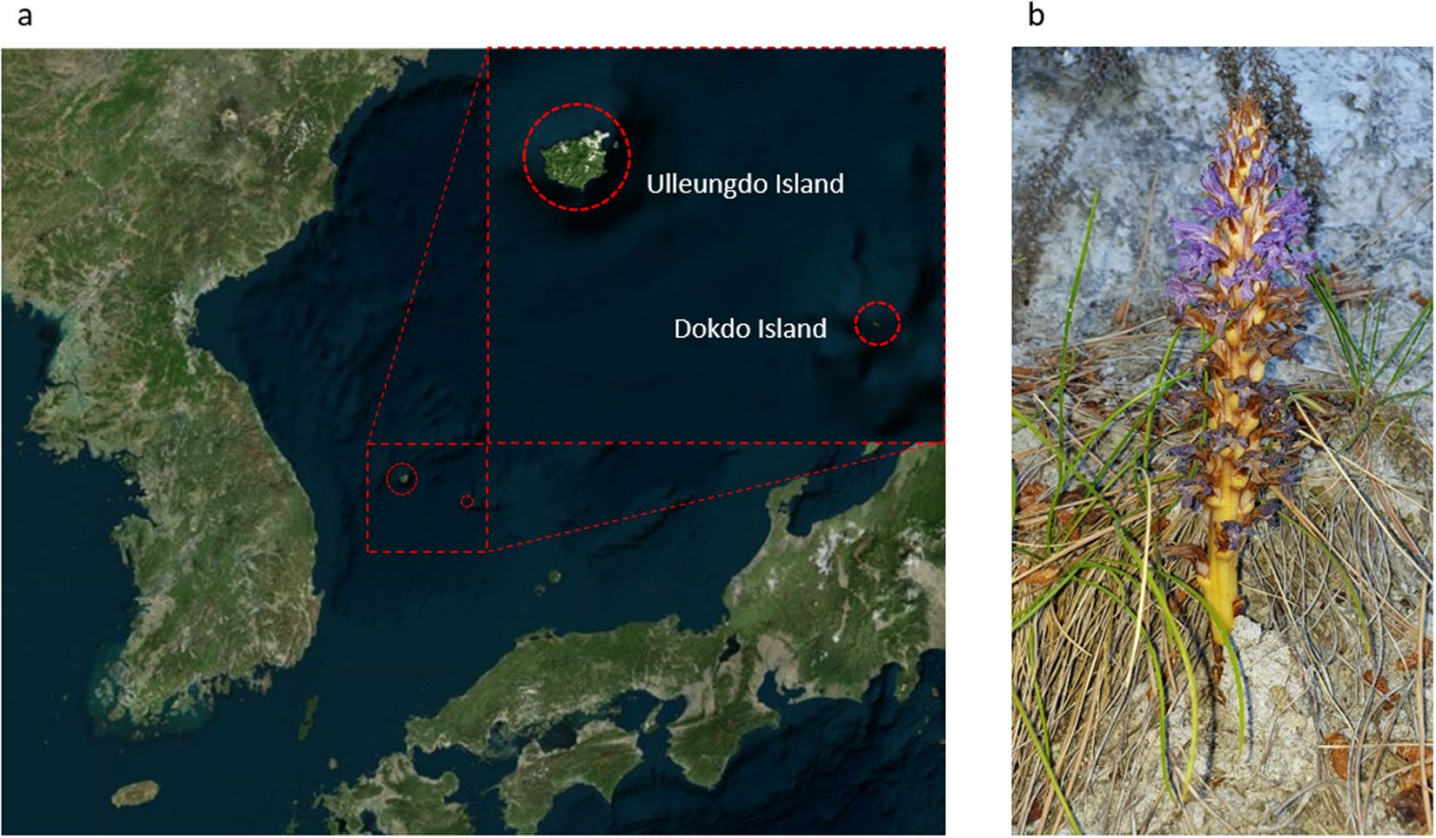
Habitats and morphology of *Orobanche coerulescens* on Korean volcanic islands. (**a**) Habitats of *O. coerulescens* on Korean volcanic islands. (**b**) Glabrous type of *O. coerulescens* collected from Ulleungdo Island.

While *O. coerulescens* is widely distributed across Eurasia, it is classified as scarce and endangered in Central Europe^8^. Likewise, there is increasing concern about its risk of extinction in Korea^9^. Notably, the species is found on the volcanic islands of Ulleungdo and Dokdo in Korea, exhibiting a distinct geographical niche compared to its presence on the Korean peninsula^10^ (Fig. 1). Interestingly, the plants on these islands are glabrous and tend to parasitize on *Artemisia japonica* rather than *Artemisia capillaris*, distinguishing them from those on the mainland counterparts^9^. Despite this ecological specialization, *O. coerulescens* faces a precarious existence due to its limited habitat range and susceptibility to external disturbances, leading to potential declines^9^. The unique geographic and ecological circumstances of *O. coerulescens* found on Ulleungdo and Dokdo Islands underscore the importance of our comprehensive genomic analysis, which aims to understand the evolution of parasitic plants and conserve this unique biodiversity.

To understand the phylogeny of Orobanchaceae and genome evolution mechanisms in parasitic plants, various studies have been conducted. These focus on aspects such as reductive evolution under relaxed selection and structural rearrangement of the genome. Specifically, several studies have targeted on constructing the chloroplast genome of Orobanchaceae^11–13^. However, given the limited scope of these studies to chloroplast genomes, there is a compelling need for complementary research focused on the nuclear genome.

**Fig. 2:**
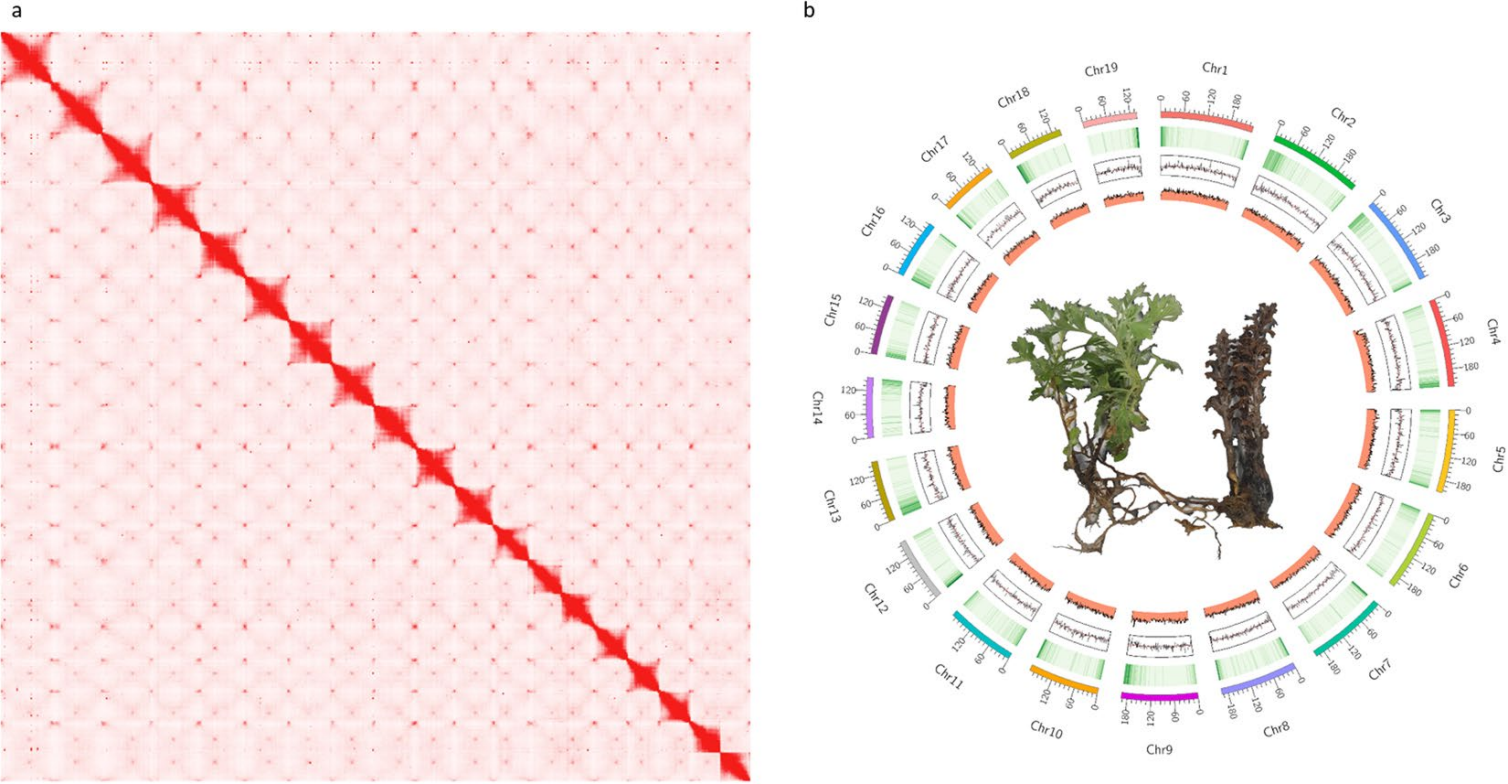
Contact map and overview of the *O. coerulescens* genome. (**a**) Contact map of 19 chromosome-level genome. (**b**) Circos plot features from the outermost to the innermost circles indicate gene density, GC content, and density of repeat elements. The figure depicts the host plant, *Artemisia japonica*, and the holoparasitic plant, *O. coerulescens*.

With the ongoing advancements in sequencing technologies, we utilized Oxford Nanopore Technologies (ONT), Illumina, and Hi-C methods to assemble a chromosomal-level genome of *O. coerulescens* (Fig. 2). The final assembly consists of 19 chromosome-level sequences and spans a total length of 3,648 Mb, with an N50 value of 195 Mb. Repeat sequences were found to constitute 86.3% of the entire genome. A total of 29,395 protein-coding genes were annotated with an average length of 4,849 bp, composed of 159,445 exons. This high-quality genome will serve as a valuable genetic resource for understanding the parasitic plant evolutionary mechanisms within Orobanchaceae and for conserving the unique biodiversity of endemics on Korean volcanic islands.

## Methods

### Sampling and sequencing

*O. coerulescens* was collected from Ulleungdo Island, Korea (37.544826 N, 130.908320E) (Fig. 1). All experimental procedures were conducted in compliance with national and international guidelines. The plant materials used was sourced from adult plants and adhered to with Korean guidelines.

The stem of *O. coerulescens* was used to obtain high molecular weight (HMW) DNA following a nuclei isolation method^14^ and genomic DNA was extracted using a modified cetyltrimethylammonium bromide (CTAB) protocol^15^ in the presence of 2% polyvinylpyrrolidone (1% of molecular weight 10,000 and 1% of molecular weight 40,000) (Sigma-Aldrich, Burlington, MA, USA). For Nanopore sequencing, short genomic fragments were removed using a Short Read Eliminator Kit (Circulomics, Baltimore, MD, USA) and the library was prepared using the ONT 1D ligation Sequencing kit (SQK-LSK109, Oxford Nanopore Technologies, Oxford, UK) with the native barcoding expansion kit (EXPNBD104) in accordance with the manufacturer’s protocol. The library was loaded onto a MinION flow cell FLO-MIN106 (Oxford Nanopore Technologies) and PromethION flow cell FLO-PRO002 (Oxford Nanopore Technologies) and sequencing was run on a MinION MK1b and PromethION. A total of long-read DNA data, 354,136,057,138 bp in length, was generated with an N50 value of 33,824 bp and a sequencing depth of 97x. To generate short high-quality sequences, we also used an Illumina platform with TruSeq DNA PCR-Free DNA library (Illumina, San Diego, CA, USA) and then paired-end DNA library was sequenced in the Illumina NovaSeq. 6000 with the length of 151 bp and the depth of 82x (Table S1).

We additionally employed Hi-C data for chromosome-level genome assembly. The Hi-C library was constructed as follows. Ground flower, stem, and root were mixed with 1% formaldehyde for fixing chromatin, and then the nuclei were isolated following a nuclei isolation method^14^. Fixed chromatin was digested with HindIII-HF (New England BioLabs), and we filled the 5′ overhangs with nucleotides and biotin-14-dCTP (Invitrogen) and ligated free blunt ends. After ligation, we purified DNA and removed biotin from unligated DNA ends. Fragmentation and size selection were performed to shear the Hi-C DNA. Hi-C library preparation was performed using the ThruPLEX® DNA-seq Kit (Takara Bio USA, Inc, Mountain View, CA, USA) and sequenced in Illumina NovaSeq. 6000 (Illumina) with the length of 151 bp paired-end reads. The Hi-C data were sequenced in 212,809,764,588 bp with 1,409,336,338 reads (Table S1).

Total RNA was isolated using TRIzol Reagent (Invitrogen) from three tissues of the same *O. coerulescens*-flower, stem, and root-following the manufacturer’s protocol. Messenger RNA (mRNA) was isolated using the Magnosphere™ UltraPure mRNA purification kit (Takara) according to the manufacturer’s instructions. The cDNA library was prepared using the cDNA-PCR Sequencing Kit (SQK-PCS109, Oxford Nanopore Technologies) with the PCR Barcoding Kit (SQK-PBK004, Oxford Nanopore Technologies) in accordance with the manufacturer’s protocol. The library was loaded onto the MinION flow cell FLO-MIN106 (Oxford Nanopore Technologies) and then sequenced on MinION Mk1b. We also used an Illumina platform data using TruSeq Stranded mRNA Prep kit for constructing cDNA library and Illumina NovaSeq. 6000 (Illumina) with the length of 101 bp paired-end reads. The results of RNA sequencing data were described in Table S1.

### Chromosome level genome assembly

*De novo* assembly was performed using a combination of NextDenovo v2.4.0^16^ and NextPolish^17^ with default parameters, utilizing ONT long reads and Illumina short reads. To obtain a high-quality chromosome-level genome, we applied the Juicer 1.6^18^ and 3D-DNA v180419 pipeline^19^ in diploid mode with default parameters. The assembly was further refined and manually curated using a contactmap generated with Juicebox v1.13.01^20^. The final assembly comprised 19 chromosomes, spanning a total length of 3,648,003,138 bp and featuring an N50 value of 195,125,857 bp (Fig. 2 and Table 1).

**Table 1:** Genome assembly statistics.

	Assembly statistics
Genome size (bp)	3,648,123,009
Number of chromsome-scale sequences	19
Number of sequences	1,943
Minimum sequence length (bp)	1,000
N50 (bp)	195,125,857
Maximum sequence length (bp)	237,001,669
GC content of the genome (%)	41.04%
	**BUSCO analysis**
Complete	1,323 (82.0%)
Complete and single copy	1,267 (78.5%)
Complete and duplicated	56 (3.5%)
Fragmented	21 (1.3%)
Missing	270 (16.7%)

### Repeat and protein gene annotation

Repeat annotation in the *O. coerulescens* genome was conducted through two different algorithms: *de novo* and homology-based methods. For *de novo* repeat identification, a custom repeat element library was constructed using RepeatModeler 2.0.3^21^ with the long terminal repeat (LTR) structural discovery pipeline option, as well as RECON 1.08^22^, RepeatScout 1.0.6^23^ and TRF 4.09^24^, utilizing the RMBLAST 2.10.0 search engine. Additionally, MITETracker^25^ was used to detect miniature inverted-repeat transposable element (MITE) repeat sequences. This custom library was then augmented with data from Repbase 20181026^26^ and Dfam 3.6^27^ databases to identify repeat sequences homologous to previously known repeat elements. Based on this comprehensive library, RepeatMasker 4.1.4^28^ was applied to annotate the repeat elements. In the constructed genome, a total of 3,147,830,327 bp, accounting for 86.29%, was composed of repeat sequences. Of these, retroelements such as short interspersed nuclear elements (SINEs), long interspersed nuclear elements (LINEs), and LTRs accounted for 0.02%, 1.35%, and 55.85%, respectively, while DNA transposons constituted 5.52% (Table 2).

**Table 2:** Statistics of repetitive elements.

Type	# of elements	length	% of sequence
Retroelements	2,503,976	2,087,694,629	57.23%
SINEs	7,260	872,464	0.02%
LINEs	81,189	49,262,217	1.35%
LTR elements	2,415,527	2,037,559,948	55.85%
DNA transposons	459,207	201,508,109	5.52%
Rolling-circles	18,244	10,291,521	0.28%
Small RNA	8,102	5,234,904	0.14%
Satellites	187	17,447	0.00%
Simple repeats	291,412	13,639,164	0.37%
Low complexity	38,213	2,129,551	0.06%
Unclassified	1,994,585	827,315,002	22.68%
Total	5,313,926	3,147,830,327	86.29%

To annotate protein-coding genes, we first assembled transcriptomes by hybrid approach of long reads and short reads from three tissues. Reads from Illumina and Nanopore were trimmed using Trimmomatic v0.39^29^ and Porechop v0.24^30^, respectively. The cleaned Illumina reads were then mapped to the *O. coerulescens* genome using STAR and assembled alongside the cleaned Nanopore reads through StringTie v2.2.1^31^. Extracted transcriptomes were processed with TransDecoder v5.5.0^28^ using default settings. Gene structure prediction was carried out using the MAKER v3.01.04^32^ pipeline, which integrates three different methods: *de novo*-based, transcript evidence-based, and homology-based. *De novo*-based method employed Augustus v3.4.0^33,34^, BRAKER v2.1.6^35–39^, GeneMark-ET v3.62^40^, and SNAP v2.51.7^41^ for gene model annotation. Transcript evidence-based method utilizing Exonerate applied to expressed sequence tags (EST) data, while homology-based method sourced data for *Sesamum indicum* (GCF_000512975.1) and *Striga asiatica* (GCA_008636005.1) from National Center for Biotechnology Information (NCBI). Using default settings, MAKER merged the outputs from these three methods to generate weighted consensus gene structures, resulting in the acquisition of 29,395 gene composed of 159,445 exons. The annotated genes included 4,203 mono-exon genes and 25,192 multi-exon genes, with an average gene length of 4,849 bp (Table 3).

**Table 3:** Statistics of protein coding gene annotation.

Category	Value
Number of total genes	29,395
Average gene Length	4,849 bp
Number of total exons	159,445
Number of mono-exon genes	4,203
Number of multi-exon genes	25,192
Average exon length	204 bp
Number of total introns	145,775

## Data Records

The genome assembly was deposited with Genbank accession number GCA_033398695.1^42^.

The whole genome sequencing DNA data were deposited with accession number SRR26425285, SRR26465287, SRR26492730, SRR26538103, SRR26538104, SRR26542269, SRR26588572, SRR26588573, SRR26588574, SRR26588624, SRR26588625, SRR26622679, SRR26661892 and SRR26661893 under Sequence Read Archive SRP467180^43^.

The transcriptome data of 3 tissues were deposited with accession numbers SRR26426871, SRR26426870, SRR26426869, SRR26503964, SRR26503965 and SRR26503966 under Sequence Read Archive SRP467180^43^.

The Hi-C data were deposited with accession number SRR26462611, SRR26522788, SRR26522789 and SRR26622680 under Sequence Read Archive SRP467180^43^.

All results from genome annotation are available in figshare^44^.

## Technical Validation

### Evaluating genome assembly

In order to assess the quality of chromosome-level genome of *O. coerulescens*, Benchmarking Universal Single-Copy Orthologs (BUSCO) v5.2.1^45^ analysis was performed using the embryophyta_odb10 database, which includes 1,614 single-copy genes. The BUSCO analysis revealed 1,267 complete single-copies and 56 duplicates, accounting for 82.0% completeness, along with 21 fragmented and 270 missing genes (Table 1). Additionally, we carried out BUSCO analysis on the protein-coding genes. Illumina short reads were subsequently realigned to the genome assembly using BWA v0.7.17^46^, yielding a mapping rate of 99.32%. We measured the quality vale (QV) and completeness using Merqury v1.3^47^ with k-mer database, resulting in QV value of 29.03 and 92.0% respectively.

### Gene prediction and annotation validation

To evaluate the gene annotation, protein sequences were searched against reference sequences from *S. lycopersicum*, *A. thaliana*, as well as the NCBI plant RefSeq and the Swiss-Prot databases. The BLASTx v2.8.1^48^ was utilized with an e-value threshold below 1e-05 and a query coverage threshold above 40%. As a result, out of a total of 29,395 sequences, successfully matches were found for 24,706 sequences against *S. lycopersicum*, 23,590 sequences against *A. thaliana*, and 29,699 sequences against the combined NCBI and Swiss-Prot databases. Further annotation for motifs, domains and Gene Ontology (GO) was performed using InterProSCan v5.60–92^49^. This resulted in the identification of domains and motifs in 24,252 proteins according to the InterPro database, while 16,681 proteins were annotated with GO terms.

### Supplementary information

Supplementary_Tables

## Data Availability

The data analyses were performed according to the manuals and protocols provided by the developers of the respective bioinformatics tools and no custom code was used in the execution of this study. All software and codes used in this work are publicly accessible, and their corresponding versions are specified in Methods section.
